# Descriptive phenomenology study of the reasons for the low uptake of free health service package among type II diabetic patients

**DOI:** 10.1186/s12913-022-08953-9

**Published:** 2022-12-20

**Authors:** Mostafa Amini-Rarani, Saeed Karimi, Laleh Gharacheh

**Affiliations:** 1 Social Determinants of Health Research Center, Isfahan University of Medical Sciences, Isfahan, Iran; 2 Health Management and Economics Research Center, Isfahan University of Medical Sciences, Isfahan, Iran; 3 Student Research Committee, School of Management and Medical Sciences, Isfahan University of Medical Sciences, Isfahan, Iran

**Keywords:** Diabetes, Low uptake, Health service package, Phenomenology study

## Abstract

**Background:**

Although several diabetes management and control programs are introduced in Iran, rate of using such cares in patients with Type II Diabetic in Ahvaz is low and they show no tendency for receiving free diabetes service package. The aim of this study was to identify reasons behind low uptake of free health service package among T2DM patients in Ahvaz, Iran.

**Methods:**

This descriptive phenomenology study based on semi-structured guided interviews of patients with Type II Diabetic in Ahvaz, was carried out in the year 2021. Through purposeful sampling, 495 patients with diabetics who not received health services package more than 6 months were interviewed until the data saturation. The gathered data were analyzed through conventional content analysis.

**Results:**

Reasons were categorized into three themes which include 13 subthemes and 57 codes. Themes included individual, accessibility, and structural factors. Besides, subthemes were lack of awareness, poor health literacy, adverse patients experience, difficulties to use services, verbal miscommunication cultural barriers, low trust, geographic barriers, time barriers, financial difficulties, lack of human resources, poor service delivery, and organizational factors were as barriers to participation.

**Conclusion:**

Regarding individual level, there is a need for further training of diabetic patients. Besides, for accessibility and structural factors Iranian healthcare system needs a comprehensive integrated care for the management of diabetes, this underlines the collaboration for improving patients' uptake of free health service package.

## Background

Type II Diabetes Mellitus (T2DM) is one of the common metabolic disorders with increasing prevalence [1]. Diabetes is the fourth leading cause of death in most countries [2]. Diabetes has the highest Global Burden of Disease (GBD) of non-communicable diseases)NCDs( [3]. Just over 1 in 10 in the world have diabetes[4]. Diabetes is the main focus of the Global Non-Communicable Diseases Action Plan [5]. There are approximately 366 million individuals with diabetes and it is estimated that 552 million will be affected by 2030 [6].

Diabetes has the highest increase in Daly's rate among the NCDs [7]. Type II diabetes is diagnosed later than type 1 diabetes. Late diagnosis or non-diagnosis of type II diabetes can increase the risk of complications and can lead to serious damage to the body systems and cause death [8]. Diabetes has many complications, including retinopathy, neuropathy, nephropathy, coronary artery disease, peripheral vascular, and cerebrovascular [9]. The cost of diabetic patients is 1.5–2.5 times of total health budget [10]. Through care, the dangerous complications of diabetes can prevent or delay and costs and mortalities can reduce [11]. given that the control of the disease requires patient participation, achieving this purposes requires the active engagement of patients with healthcare professionals According to the latest reports, the prevalence of diabetes in Iran is 10.6 (11.2% in women and 9.8% in men) and this disease is the cause of two percent of all deaths in Iran [8]. Iran provides free services to diabetic patients in health centers through Iran's Package of Essential No Communicable Diseases program (IraPEN) [12]. Package of essential Non-communicable Diseases (NCD) interventions is part of the national health transformation plan, launched in the year 2014 by the Ministry of Health and Medical Education, to provide universal health coverage, including access to NCD prevention and care, as well as health services [2]. In this regard, on time attendance to health centers for care and control of blood sugar are examples of active participation that in some cases is not done properly [4, 13].

Research has shown that few individuals with diabetes go to health centers for free services [14–21]. Recently, despite great attention has been paid to the development of comprehensive health service centers in Iran, the number of individuals using the services of comprehensive health centers has decreased [22]. According to the report of the Diabetes Research Center and the Iranian Diabetes Association in the year 2020, Khuzestan is the second in the high prevalence of diabetes in the Iran. The desire of the individuals in Khuzestan for sweets is higher than other provinces due to the hot days of the years [23, 24]. According to the Non-Communicable Diseases Research Center (STEPs) in 2016, the prevalence of diabetes in Khuzestan in women was 183,256 (13.9%) and in men was 189,204 (14. 1%). According to the national average, the prevalence of diabetes is 10.6%, this way, the situation of Khuzestan is higher than the national average of diabetes [8]. Receiving continuous services in diabetic patients can reduce the risk of various complications such as Diabetic Foot Ulcers, Cardiac and ocular coronary artery, peripheral vascular disease, cerebrovascular disease, and death from diabetes [25–27]. Constant cares and routine controls are essential to prevent possible complications in diabetic patients. These pre-complication cares and controls can reduce the cost of treatments and decrease health system expenditure for diabetes. Package of essential NCD (PEN) interventions for primary health care IraPEN is part of the national health transformation plan (free of charge and relatively good physical access) provides NCD prevention and care services for diabetic patients, through comprehensive health centers. Nevertheless, according to the authentic statistics the rate of using such cares in patients with Type II Diabetic in Ahvaz, Iran is low and they disinterest in receiving free diabetes service package [28]. Therefore, this study endeavored to identify reasons why T2DM patient's low uptake of free health service package in Ahvaz, Iran. This study concentrates on the literature by exploring how diabetic's patients become motivated and interested to seek free health services.

## Methods

### Study design

We employed a descriptive phenomenology based on semi-structured guided interviews of T2DM patients in Ahvaz in the year 2021. In this study, 495 T2DM patients in Ahvaz were interviewed to deepening our understanding of reasons behind low uptake of diabetic's health services package with T2DM patients who have had at least one year of experience with type II diabetes and they did not use the free health package services more than 6 months in comprehensive health centers.

### Phenomenological method

A descriptive phenomenological study was conducted to arrive at a detailed understanding of the reasons for the low uptake of free health service package among T2DM Patients. Descriptive phenomenology involves the exploration and analysis of a particular phenomenon to arrive at subjective, experiences of the participant [29].

This approach is used when little is known about an issue, and the aim of the study was to explicate and understand the phenomenon from the perspective of those directly involved in it [30]. The method used in this study was Giorgi’s descriptive phenomenological method. It consists of five main steps [31], designed to condense the themes that make up the meaning units of the phenomenon in question. The first step was considered the phenomenological attitude in the data analysis for the researcher. The second step required the researcher to read and re-read the entire description of the person’s interview to develop a sense of the whole experience and become familiar with the data. The third step involved identifying units of general meaning that described participant’s experience of the phenomenon of interest The fourth step required the researcher to transform each unit of general meaning originally expressed in the participants’ own words, into statements, without changing the meaning content. The fifth step involved synthesizing the insights into a descriptive structure of the meaning of the experience [32].

### Study setting

Ahvaz is a city in the southwest of Iran and the capital of Khuzestan and situated on both banks of Karun River. The river divides the city into two districts (East and west of Ahvaz), Ahvaz's population is about 2,000,000. It is home to Persians, Arabs, Bakhtiaris, Dezfulis, Shushtaris, and others. Languages spoken in the area include Persian and Arabic, as well as dialects of Luri (Bakhtiari), Dezfuli, Shushtari, and others.

**Fig. 1:**
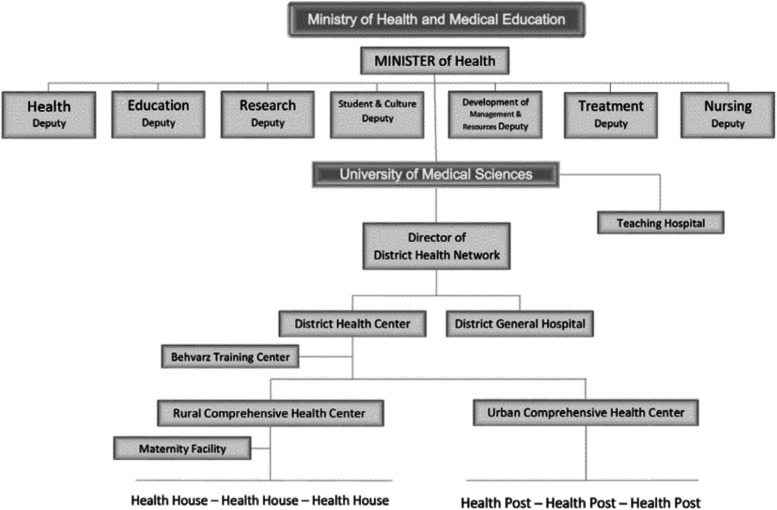
Health System Network in IRAN

Healthcare system in Ahvaz is governed by Iran's Ministry of Health, Treatment, and Medical Education. The ministry also regulates medical services in Ahvaz which are offered based on three systems including the public-governmental system, the private sector, and NGOs. Healthcare and public health services are provided through a strong network offering different levels of healthcare services, namely primary health care, secondary healthcare, and tertiary health care to fulfill the goal. The health network system is designed to ensure an optimal healthcare in urban and rural areas. The medical facilities that are ruled based on the public-governmental system carry out their activities and offer services under the direct management of medical universities. The public sector provides primary, secondary, and tertiary health services considering primary healthcare. Public hospitals and clinics are financed by governmental budget and the services at this sector are covered by different health insurances available in Iran (Fig. 1); this way, patients receive medical services at the possible lowest price. To eliminate health disparities, advance prevention, and strengthen primary care access, an extensive healthcare network offers primary healthcare in rural and urban areas. Rural healthcare networks include a number of “Health Houses” and a district health center. Health houses are the periphery and basic unit of Iranian PHC system and the first point of contact between the primary healthcare system and the community in rural areas. They are managed by a health center, located at the regulatory district, which is affiliated to the main medical university of province. These health houses provide preventive health care services such as nutrition, diabetic control, medical screening. A similar system provides primary care in urban and suburban areas by health posts and urban health centers of the major cities, which often act as provincial capitals. To provide healthcare on an equitable basis and to ensure better quality health services, the system also follows a well-planned referral care mechanism where family physicians, covering a population of about 2000 to 4000 in their catchment areas, provide primary healthcare services and if required refer the patients to the secondary and tertiary healthcare centers.

In addition to the public-governmental facilities, private medical facilities including for-profit hospitals and clinics, however, supervised by medical universities, operate independently to deliver effective and efficient care. The private sector in Ahvaz mainly focuses on secondary and tertiary healthcare in urban areas.

In urban area, Ahvaz health networks manages 19 East Ahvaz Urban Comprehensive Health Centers and 14 West Ahvaz Urban Comprehensive Health Centers [8, 23].

### Samples

This study endeavored to find the relevant interviewees from a sampling frame among all the T2DM patients who covered by comprehensive health centers. Samples were selected from 19 comprehensive health centers in the west and 14 comprehensive health centers in the east of Ahvaz. Each center has covered a population of at least 35,000 to 40,000. A total of 495 eligible T2DM patients were interviewed conducted as long as reaching a saturation point, in which exceeding no more data was needed (15 patients from each comprehensive health center). The purposeful sampling with maximum variation approach was applied to select different participants in terms of location, cultural, social, economic, racial and linguistic, age, education, nationality and diabetic status.

The inclusion criteria were as follows:

- 1. T2DM patients who did not use the free health package services more than 6 months in comprehensive health centers,
- 2. Being a Type II Diabetic patient for at least 1 year prior to the interview,
- 3. Being over 18 years old,
- 4. Being mentally and physically able to participate in the face-to-face interviews.

In each comprehensive health centers, an informed staff helped us to find a list of eligible T2DM patients. Data collection was carried out via semi-structured interviews. A researcher (LG) collaborated with 4 research assistants who had experience in qualitative studies were conducted interviewers. Interviews were conducted from the years 2021 to 2022.

Postal address of diabetic’s patients who not received health services package in comprehensive health services more than 6 months were taken from patients' electronic health records. Then, after setting an appointment with participants, face-to-face interviews were conducted in any places where they suggested.

The interviews were short and lasted at most for 20 min and continued until data saturation.

The interviews prompts are: "Why did not you go to the health center for the past few months to receive free diabetics services?" or “what problems do you faced for using free health service package provided in health centers for T2DM patients?”,

This procedure was conducted in 5 months. With the consent of the participants, all interviews were recorded through a tape recorder, and then transcribed verbatim. These attempts were made to conduct the interviews without bias and to write only the gist.

### Data analysis

This study used conventional content analysis [33] with an inductive data-driven process coding and theme development for the analysis of the transcribed interviews [34].

The interviews were reviewed several times to infer a list of inductive codes and themes. The conventional content analysis was conducted as follows:

In the first stage, authors independently coded the collected data. They read and re-read the transcriptions with the aim of becoming familiar with the data.

The second stage includes primary themes were extracted from the obtained data and reviewed by the team members. The team members held a meeting to elaborate on controversial points and reach an agreement on emerging themes. They continued discussion until addressing the controversies. This way, themes and sub-themes were identified.

Finally, team members reviewed, modified, and collated coded statements. Transcribed documents were managed and analyzed using MAXQDA Standard 2020.

### Trustworthiness

Trustworthiness was ensured using the criteria proposed by Lincoln and Guba [35, 36].

To confirm credibility, data collection, coding, and analysis were done in 10 months. Moreover, the researcher took notes within interviews for important points. The notes were taken into account during the coding process. The texts also contained some embedded quotations that helped ensure the transferability, which is directly dependent on external validity. Also, data collection and data analysis were conducted simultaneously for improving transferability. Maintaining an audit trail of the study processes established dependability. In this approach, the authors, along with an external auditor, provided complimentary comments, crosschecked, examined inconsistencies, and addressed them for reaching an agreement. For confirmability improvement, the research team did not allow their values and inclinations to bias the study and the findings. In other words, bracketing- in which we set aside our experiences, as much as possible- was assured.

## Results

As to the descriptive analysis, as Table 1 shows, 495 participants were selected from among who low uptake of the health service package from healthcare centers. Among them, about 52.5% was men and 47.5% was women, participants were on average more than 58 years old. Most participants were married and diploma.

**Table 1:** Demographic features of participants in the study

Features	Mean (SD)/Frequency (%)
**Sex**	
Men	260(52.5%)
Women	235(47.5%)
**Age**	58.61(11.96)
**Duration of diabetes **	8.90(4.62)
**Education**	
primary school	79(16%)
Less than a high school diploma	62(12.5%)
diploma	184(37.2%)
Bachelor	114(23%)
Masters	40(8.1%)
Doctorate	16(3.2%)
**Marital Status**	
Married	488(98.9)
Single	7(1.1%)
**Total**	495

Reasons for low uptake of free health service package were categorized into three themes which include 13 subthemes and 57 codes, which are shown in Table 2.

**Table 2:** Theme, subthemes and codes of reasons for the low use of free health service package among T2DM patients

Theme	Subthemes	Code
**Individual factors**	Lack of awareness	- About diabetic service package - About times of service delivery - About location of health centers
	Poor health literacy	- Low capacity to reading diabetes healthcare information - Low capacity to obtain diabetes related health information - Low capacity to process diabetes related health information - Low capacity to understand diabetes related health information
	Adverse patients experience	- Delay in receiving diabetic services -Ineffective care - Lack of follow-up - Poor doctor- patient’s relationship (D.P.R) - Giving misleading information - Too long waiting list - Crowded health center - Inadequate visiting time - Poor service - Personnel bad behavior
	Difficulties to use services	- Aging - Physical disability - Mental disorder - Illness
	Verbal dis-communication	- Language differences - Use of scientific terminology by providers - Different mode of speaking
	Cultural barriers	- Poor culture - Adherence to wrong ancient culture - Anti-medicine (not believe to modern medicine) - Using fake traditional medicine -Racial barriers (conflicting values) -Attitude to the disease - Poor acceptance of illness
	Low trust	-Lack of trust to prescriptions -low trust to public health services - Lack of trust to healthcare worker’s recommendations - Lack of trust to health worker’s information
**Accessibility**	Geographic barriers	- Long distance between home and health centers - Difficult transportation - Bad location health centers
	Time barriers	- Long travel time -Long waiting time -Inappropriate health center working time
	Financial difficulties	-Cost of medicine -Cost of transportation -High cost of prolonged treatment - Unaffordability of poor diabetic patients
	Lack of human resources	- Lack of health providers - Lack of physician - Lack of specialist -Part time physicians
**Structural**	Poor service delivery	- low quality of services - No available clinical or para clinical services - Poor consultation - Staff absenteeism at work
	Organizational factors	- Referral system problems - Out‐of‐date equipment and facilities‏ - Unmotivated staff - Low educated personnel for diabetic services

The T2DM patients emphasized that three factors individual, accessibility and structural factors were reasons for low uptake of free health service package in urban healthcare centers.

### Individual factors

#### Lack of awareness

Lack of awareness is one of the reasons for low uptake which include lack of awareness diabetic service package, lack of awareness times of service delivery, and lack of awareness location of health centers. In this regard one of the participants stated that "I had no information about services for patients with diabetes in health centers and I do not know how often I should be visited by health staff and family physicians "(Participant 11).

Another patient said that "I did not know about working times and location of health centers "(Participant 73).

### Poor health Literacy

Poor health literacy is another reason for low uptake which means patients low capacity to reading diabetes healthcare information of brochures, guides… low capacity to obtain diabetes related health information. Low capacity to process diabetes related health information. Low capacity to understand diabetes related health information.

Participant said that: "When I go to the centers and the experts talk about diabetes, most of the time I cannot the expert message talking about or if they give me a pamphlet or brochure for guidance, I will not be able to read them." (Participant 51).

One patient stated that “I do not know the amount of sugar controlled, I think this wound on my foot is not due to blood sugar” (Participant 192).

### Adverse patient's experience

Patients emphasized the main reasons for low uptake of health Centers is Adverse Previous experience. Participant stated that "when I go to health centers, I have to wait a long time due to the large population and the lack of staff to receive services, and this is boring for me and lot of time is wasted " (Participant 42). Crowded health center and too long waiting list and delay in receiving diabetic services is long is another reason.

### Lack of follow-up

Patients should be visited and cared every three months for blood sugar control, but some patients do not refer timely. One of the patients in this regard said that “we do not know when the doctor is present in the center and the health worker does not inform us” (Participan 61).

### Poor doctor- patient’s relationship (D.P.R)

It seems that the first step in providing health services is effective communication between physician and patient, but because of the temporary nature of family physicians and unfamiliarity with local language of patients, this communication is poor.

As to patients who have not received suitable services in previous visit to the centers, one of the patients said that “The doctor did not pay enough attention to my biography" (Participant 92).

Another participant said that "My blood sugar did not determine correctly by Providers "(Participan 101).

### Difficulties to use services

Patients have problem referring to health centers because of growing older or due to their physical disability such as musculoskeletal, vision problems, illness, mental disorder, and aging. They have problems and need others help. In this regard, a patient said;

In this regard a participant stated that “I cannot go alone because I am visually impaired and my family cannot take me to the center. [in fact,] they are busy” (Participan 205).

### Verbal dis-communication

Communication is the important facet of health care provision [37].

Language barriers in healthcare include (language differences, use of academic terminology, different mode of speaking) lead to miscommunication between the medical professionals and patient, reducing both parties' satisfaction and decreasing the quality of healthcare delivery and patient safety. Besides, effective communication allows patients to feel comfortable talking to their doctors and understand vital information about their health.

Most individuals in Ahvaz are Arabs who have difficulty in communicate in Persian.

A participant said that “I cannot speak Persian so When I go to the health center, I cannot have a communication with providers” (Participan 227).

Another patient said that "When I go to the health center, I don't feel comfortable to providers or doctor due to the high frequency of professional words by providers and I do not understand their sentences; thus, going to these centers is useless to me" (Participan 312).

### Cultural barriers

Some female patients because of prejudice of their husbands, tradition and local customs, cannot refer health center alone. A participant said that “my husband is prejudice and do not agree with my referring health center alone”(Participan 347). Another participant is of the opinion that “I prefer local and herbal medicine as treatment because they are safer and more secure than prescription drugs” (Participan 319).

A participant also said that “because I use narcotic again, my sugar level has decreased and I do have diabetes anymore and do not need to take drug” (participant 412).

### Low trust

Patients need to be able to trust health providers with their lives and health. Patients with high levels of health care-related distrust avoid health care. Patients with high levels of healthcare-related distrust avoid healthcare. Low trust in healthcare in this study includes lack of trust to prescriptions, low trust to public health services, lack of trust to healthcare worker's recommendations, lack of trust to health worker’s information. Participant 198 said that "in my previous visit to the center, I received that the level of personnel health information is low".

Also, participant 315 commented that "in the previous visit, health providers measured my blood sugar mistakenly and referred me immediately to the hospital, because of this mistake, I spent a lot of time and money."

### Geographic barriers

Patients in distant situations without appropriate transportation system are not able to use the health center. In this regard, a patient (Participant 392) said that" health center is far from our house and has a bad road and I don’t have personal vehicles to refer to the centers easily."

Another patient (participant 403) said that "The location of the health center is not good and it is not safe. There have been several robberies in that area."

### Time barriers

Time barriers is one of the reasons for low uptake which include: long travel time, long waiting time, and inappropriate health center working time.

Patient (Participant 276) stated that "When I go to health center to receive services. It takes a long time for me to go to health center and back, because of the crowded center and lack of staff, I have to be in waiting time in centers for an hour or two and this drives me to lose my time of day".

Another patient (Participant 332) remarked that "Regarding my occupational problems I have a limited time to visit but health services are offered exclusively during working hours in the morning."

### Financial difficulties

The problems that patients have related to the costs of medicine, transportation, and high cost of prolonged treatment may lead to low uptake of health service package. One of the participants said that "If I be referred to hospital or other premises for specialist treatment or diagnostic tests by a doctor, or another primary care health professional, I have low income to afford costs of medicine and treatment."

Besides, another patient (participant 148) said that “In the previous visit, I was referred to the hospital for treatment, but because I am unemployed, it is difficult for me to pay for medicine and treatment.

### Lack of human resources

The lack of health providers, physician of health workers has been identified as one of the constraints to uptake of T2DM patients in health centers. One of the participants said that "Two people, a midwife and the other a healthcare worker, are working in a health center and cannot address the needs of the large number of clients". A patient (Participant 401) said that “Sometimes I refer to health center, but they say we do not have doctor and I have to refer again another day, it is difficult for me, we have other things to do.”

Participant 381 said that: "The health center that is near to our house, the doctor is not there every day and access is very difficult."

### Poor service delivery

Inaccurate diagnosis, medication errors, inappropriate or unnecessary treatment, inadequate clinical facilities or practices, staff absenteeism at work are just some of the highlights from poor service delivery. 

The participant 197 said that "I think, in health centers do not provide good advice for patients with diabetes, and every time I went to the health center, I was not satisfied with the quality of advice provided by experts." Another participant 283 said that "No laboratory or clinical services are provided to patients in health centers. Sometimes the blood sugar monitor is even broken or does not show the correct blood sugar rate."

### Organizational factors

Organizational Factors is one of the reasons for low uptake which include: referral system problems, out‐of‐date equipment and facilities‏, unmotivated staff, low educated personnel or providers who lack adequate training and expertise prevail.

In this regard, participant 406 said that "I think health workers are tired and do not have motivation. Doctors also do not have enough experience and do not take work serious and I prefer not to go to the health center". Another participant 285 said that "Health centers have systemic problems. Several times I was confused and wasted time due to the problems of the health center to go to higher levels."

## Discussion

This qualitative study was done to identify the reasons why T2DM patient's low uptake of urban free health service package in Ahvaz.

Findings revealed that the 3 main themes such as Individual factor, Accessibility, structural and 13 Subthemes and 57 codes were extracted.

In the study, a wide variety of T2DM patients' reasons regarding low uptake of health service package were identified, in which many of them were similar reasons identified in other studies around the world.

These should be properly considered in the design and implementation of the free health service package plan and coordinated at different levels to ensure the proper achievement of the plan's goals and the promotion of effective health systems. Individual factors were the first obstacle in the low uptake of patients from the free health service package.

In the research done by Babaei Heydarabadia et al. demonstrated that personal factors includes low patient knowledge, poor compliance, poor patient participation, busy schedule and time constraint, physical disability as patients causes of do not attend [38].

Our findings were also consistent with those a systematic meta review by Verónica Ciro et al. has demonstrated that both individual and contextual factors such as a lack of knowledge of the existence of guidelines and negative attitudes among females are related barriers in diabetic care facilitators [39]. Sharifi et al. also in qualitative study pointed individual factors include physical abilities is causes for patients’ non-participation in self-care training program [40]. Besides, in study of Bidarpoor et al. inadequate informing of location of health centers to community, was the barriers to low uptake of patients. [41]. Health literacy is the degree to which individuals have the capacity to obtain, process, and understand basic health information needed to make proper health decisions [42].

Regarding the health literacy barriers, in Sharifi study attitude to health and education are reasons for non-participation in a self-care training program for diabetic patients [37].

Low capacity to reading diabetes healthcare information in brochures, guides, and poor health literacy is one of reasons for low uptake of free health service package. In the research by Momeni et al. it was shown that health literacy is not high in Iranian diabetic patients and is rather inadequate in most of them in both genders. Diabetic women have somewhat lower health literacy compared to diabetic men [43].

According to the US Department of Education, only 12% of English-speaking adults in the US have proficient health literacy skills. Approximately 45% of high school graduates in the US have limited health literacy [44, 45]. In Meares et al.’s study in Chicago, it was revealed that low health literacy continues to be a barrier to obtaining high-quality healthcare for many individuals from disadvantaged groups [46].

Saeed et al. found health literacy was inadequate in 67.15% of patients; the majority of the diabetes patients had poor health literacy [47]. also, in study by Hussein et al., inadequate health literacy was revealed in most diabetic patients [48].

Quartuccio et al. study showed that poor health literacy was higher in older male diabetic patients compared to healthy older men, [49]. Most diabetic patients have poor health literacy. In Ilam, Iran, only 27.2% of diabetic patients had adequate health literacy [50, 51].

In this study adverse patient's experience was another barrier for T2DM patients’ low uptake of free health Service package. Another study found experienced by participants related to insufficient GP and specialist consultation time were common challenges. Also shown that poor communication was a major barrier to good health care. They saw limited time and human resources as ‘significant barriers’. They observed that specialists are lack sufficient time to provide thorough information [52]. also in other research, the factors contributing to non-compliance among diabetics attending primary health centers was physician–patient relationship [53]. In Bidarpour et al. study, shown that inappropriate attitude of employees and time-consuming services were mentioned as reasons of non-attendance of households in the comprehensive centers of health services [38]. in other study by Mohseni et al. found that poor service delivery was barriers of managing diabetes in Iran [24].

In this study, another reason for patients’ low uptake of free health service package was physical disability. In Saudi Arabia, Alkawai et al. found that the majority of patients with physical disability require assistance and cannot move around independently in the healthcare facilities. Nearly half of these patients face several challenges in accessing healthcare services and are unsatisfied with the services received [54].

In study by Tesfaye et al. in Hawassa confirm that accessibility to healthcare services among people with disabilities was poor [55].

A mixed methods systematic review study by Edwards et al. shown that people with disabilities have lower uptake of preventive services, compared with people without disabilities [56]. Language barriers was another reason from the patients’ views.

Because, the mother language in Ahvaz is Arabic, patient with Persian language cannot appropriately communicate in Ahvaz.

According to the results of Shamsi et al. A systematic review study, shown that language barriers in healthcare lead to miscommunication between the medical professional and patient, reducing both parties' satisfaction and decreasing the quality of healthcare delivery and patient safety [57].

In study finding by Ali et al., language barriers were identified as the biggest obstacles in good providing to patients with limited English proficiency [58]. The results of study shown that cultural barriers are reasons of T2DM patients’ low uptake of free health service package. These reasons are diverse and include poor culture, adherence to wrong ancient culture, anti-medicine means not believe to modern medicine, using fake traditional medicine, racial barriers or conflicting values. These barriers can limit uptake to healthcare services in various forms. Individual cultural beliefs also influence the patients’ uptake of free health service package. Most moral values influence to medicine and healthcare decisions. This can be linked to a lack of information to help them understand the importance of modern medical practices.

In a study by Mogre et al. in Ghana found that cultural beliefs were one of the barriers to diabetic health care [59].

The results of study shown that cultural barriers are reasons of T2DM patients’ low uptake of free health service package. These reasons are diverse and include poor culture, adherence to wrong ancient culture, anti-medicine (means not believe to modern medicine), using fake traditional medicine, racial barriers or conflicting values. These barriers can limit uptake to healthcare services in various forms. Individual cultural beliefs also influence the patients’ uptake of free health service package. Most moral values influence to medicine and healthcare decisions. This can be linked to a lack of information to help them understand the importance of modern medical practices.

In a study by Mogre et al. in Ghana found that cultural beliefs were one of the barriers to diabetic health care [60]. In Babaei Heydarabadia’s study shown that tradition and local customs are reasons for diabetic patients not attend for scheduled appointments [38].

In this way, various studies suggested programs to help health providers better understand different cultural backgrounds, and modern healthcare practices [61].

Moreover, another influential cause, is low trust includes: lack of trust to prescriptions, to public health services, to healthcare worker’s recommendations, to health worker’s information were reasons for patients with low uptake of free health service package. In line with findings of other studies in UK and USA in primary healthcare [62, 63].

This study found that a trusted relationship between patients and health providers promotes ‘adherence to health-related interventions due to patients’ trust in providers' advice.

In Birkha¨uer’s study founded that a significant association between trust in the health care and health outcome [64]. Besides, bidarpoor study shown that low trust was important reasons of non-attendance of households in the comprehensive centers of health services [41].

In our study, Geographic barriers is one of the reasons for low uptake of free health service package which include: long Distance between home and health centers, difficult transportation, bad location health centers.

In the research by Babaei et al. shown that transportation problem is one of the reasons for non-referral of diabetic patients to medical centers [38]. Besides, in Ataur study, the majority of patients who could not see the doctor on the day of appointment mentioned unavailability of transport and forgetfulness as the main reasons [53].

In study by sharifi and bidarpoor, it was revealed that physical access and long distance from the location were important Reasons for diabetic patients [40, 41]. Another reason for low uptake in our study was time barriers which include: long travel time, long waiting time, inappropriate health center working time that this barrier has been mentioned in other studies [38, 40, 41].

In Kelly et al.’s systematic review it was revealed that relationship between travelling further and patient low uptake cannot be ruled out and should be considered within the healthcare services location debate [17]. Waiting time has been defined as the length of time between enrolling a patient on a waiting list and the period that a patient takes at each point of service before being treated [18]. In various studies shown long waiting time adversely affects the willingness of the patient to return to the health centers which will highly reduce the utilization of health services [19]. Although the health services in health centers are freely provided but in this study, we still observed additional economic burdens placed on patients and their families, for instance, cost of medicine, cost of transportation, high cost of prolonged treatment, and unaffordability of poor diabetic patients.

Various studies shown that vulnerable patient groups such as those with a low income, lack of health insurance or debt have an increased risk of forgone care [65–72].

Improving health service coverage and the highest standard of health depends on their availability, accessibility, acceptability, and quality [73].

Health workforce is made up of a range of health workers who offer healthcare services. Lack of health providers, physician, specialist and part-time physicians were another reason for low uptake. In this study, poor service delivery was one of the patients’ reasons for low uptake of free health service package which includes low-quality services, no available clinical or paraclinical services, poor consultation, and staff absenteeism at work. The unavailability of free medical tests was also found in other developing countries [74], which indicates that the policymakers should evaluate the healthcare strategies on the free health service package.

Besides, Babaei et al. reported that ‘service providers’ was one of the reasons for the absence of patients in the scheduled appointments, because of staff absenteeism at work, lack of patients’ follow-up, improper relationship between patient and doctor [35]. By the same token, Babaei emphasized on referral system problems, lack of equipment, and unmotivated staff. Poorly motivated health workers can have a negative impact on individual facilities and an entire health system. Some experts suggest that low wages and poor working conditions for healthcare workers are the causes of unmotivated staff in this part of the health system [21].

### Limitations

This study selected a high number of sample size from all urban comprehensive health centers in Ahvaz with different participants in terms of cultural, social, economic, racial, linguistic, educational and nationality; thus, the transferability of the findings is strength of this study. Nevertheless, as our findings depend more on the interviewees' lived experiences and their mentality regarding services provided by comprehensive health centers, subjectivity of findings is a limitation. However, the research team had 10-month cooperation for collecting, coding and interpreting the data, as well as, the findings were examined by an external reviewer. Also, this study used a constructivist approach rather than positivism, and so, subjectivity is unavoidable.

### Implications

According to the results of this study, it seems that by using the following policies, it is possible to T2DM patients more uptake the free service package in health centers:

- Educating patients and their families and service provider personnel, knowledge and awareness can be improved.
- Participatory decision making through patient-centered care by involving and engaging T2DM patients and families in decisions regarding diabetic healthcare services and tailored services to their preferences.
- Counseling by phone and SMS with the aim of improving and improving people's access to health and medical information related to T2DM. Using electronic referral systems with the aim of strengthening coordination, improving access, and improving the quality of health and medical services will increase the level of satisfaction and improve communication between providers and recipients of health and medical services.
- Comforting waiting area by investing in comfortable seating and decorations of waiting room. Providing special facilities near the patient's location and availability of effective communication between patient and health system.
- Hiring bilingual health staff. Employing general practitioners and health staffs who are fluent in a language that is common in region helps a patient feel comfortable enough to share important, confidential information.
- Promoting home health cares for diabetics with physical disabilities to get the care he or she needs at home while offering peace of mind to that individual's family or other loved ones.
- Revising content of training based on the community-based educational model is seeming very necessary to align the content of training to the skills health workers need.
- Improving motivation of health workers through use of both financial and nonfinancial incentives, including better salaries and decrease Health workers payment discrimination, staff recruitment and retention, streamlined management, better facilities and material resources and also acknowledge of efforts made by them.

## Conclusion

Overall, in the individual level, there is a need for further training of diabetic patients to clarify the importance of healthcare services and improve adherence to the free health service package provided in health centers.

Besides, for accessibility and structural factors Iranian healthcare system need a comprehensive integrated care for management of diabetes, in which the system improves from fragmented, disease-centered, inaccessible care to a patient-centered, holistic and continuous care.

Appropriate interventions and changes in policy implementation are required to address the health care needs and remove barriers to uptake free health care packages of the T2DM patients.

This underscores effective collaboration between researchers and policy makers, managers, physicians, health experts, other related institutions and communities for improving patients’ access to free health Service package.

## Data Availability

All data are available from the corresponding authors upon reasonable request.
